# Spike mutations contributing to the altered entry preference of SARS-CoV-2 omicron BA.1 and BA.2

**DOI:** 10.1080/22221751.2022.2117098

**Published:** 2022-09-28

**Authors:** Bingjie Hu, Jasper Fuk-Woo Chan, Huan Liu, Yuanchen Liu, Yue Chai, Jialu Shi, Huiping Shuai, Yuxin Hou, Xiner Huang, Terrence Tsz-Tai Yuen, Chaemin Yoon, Tianrenzheng Zhu, Jinjin Zhang, Wenjun Li, Anna Jinxia Zhang, Jie Zhou, Shuofeng Yuan, Bao-Zhong Zhang, Kwok-Yung Yuen, Hin Chu

**Affiliations:** a State Key Laboratory of Emerging Infectious Diseases, Carol Yu Centre for Infection, Department of Microbiology, School of Clinical Medicine, Li Ka Shing Faculty of Medicine, The University of Hong Kong, Pokfulam, Hong Kong Special Administrative Region, People’s Republic of China; b Department of Infectious Disease and Microbiology, The University of Hong Kong-Shenzhen Hospital, Shenzhen, People’s Republic of China; c Centre for Virology, Vaccinology and Therapeutics, Hong Kong Science and Technology Park, Sha Tin, Hong Kong Special Administrative Region, People’s Republic of China; d Department of Microbiology, Queen Mary Hospital, Pokfulam, Hong Kong Special Administrative Region, People’s Republic of China; e Academician Workstation of Hainan Province, Hainan Medical University-The University of Hong Kong Joint Laboratory of Tropical Infectious Diseases, Hainan Medical University, Haikou, People’s Republic of China; f Guangzhou Laboratory, Guangzhou, People’s Republic of China; g CAS Key Laboratory of Quantitative Engineering Biology, Shenzhen Institute of Synthetic Biology, Shenzhen Institutes of Advanced Technology, Chinese Academy of Sciences, Shenzhen, People’s Republic of China

**Keywords:** SARS-CoV-2, Omicron BA.1 and BA.2, pathogenesis, entry, endosomal entry pathway, fusogenicity, spike protein cleavage

## Abstract

SARS-CoV-2 B.1.1.529.1 (Omicron BA.1) emerged in November 2021 and quickly became the predominant circulating SARS-CoV-2 variant globally. Omicron BA.1 contains more than 30 mutations in the spike protein, which contribute to its altered virological features when compared to the ancestral SARS-CoV-2 or previous SARS-CoV-2 variants. Recent studies by us and others demonstrated that Omicron BA.1 is less dependent on transmembrane serine protease 2 (TMPRSS2), less efficient in spike cleavage, less fusogenic, and adopts an altered propensity to utilize the plasma membrane and endosomal pathways for virus entry. Ongoing studies suggest that these virological features of Omicron BA.1 are in part retained by the subsequent Omicron sublineages. However, the exact spike determinants that contribute to these altered features of Omicron remain incompletely understood. In this study, we investigated the spike determinants for the observed virological characteristics of Omicron. By screening for the individual changes on Omicron BA.1 and BA.2 spike, we identify that 69–70 deletion, E484A, and H655Y contribute to the reduced TMPRSS2 usage while 25–27 deletion, S375F, and T376A result in less efficient spike cleavage. Among the shared spike mutations of BA.1 and BA.2, S375F and H655Y reduce spike-mediated fusogenicity. Interestingly, the H655Y change consistently reduces serine protease usage while increases the use of endosomal proteases. In keeping with these findings, the H655Y substitution alone reduces plasma membrane entry and facilitates endosomal entry when compared to SARS-CoV-2 WT. Overall, our study identifies key changes in Omicron spike that contributes to our understanding on the virological determinant and pathogenicity of Omicron.

## Introduction

Coronavirus Disease 2019 (COVID-19) caused by severe acute respiratory syndrome coronavirus 2 (SARS-CoV-2) was first reported in late 2019 [1–3]. The virus disseminated efficiently and has resulted in a pandemic at the global scale which is still ongoing as of today [4]. Importantly, SARS-CoV-2 continues to generate new variants by acquiring mutations that alter its transmissibility, infectivity, and resistance to neutralizing/therapeutic antibodies. SARS-CoV-2 Omicron BA.1 emerged from South Africa and Botswana in November 2021 and quickly became the most prevalent SARS-CoV-2 variant worldwide due to its high transmissibility and immune evasiveness [5–9]. More recently, continuous surveillance of SARS-CoV-2 evolution revealed additional Omicron lineages, including BA.2, BA.2.12.1, BA.4 and BA.5, which have become the dominant circulating SARS-CoV-2 variants [10,11].

Omicron BA.1 contains a large number of changes in comparison with the ancestral SARS-CoV-2, particularly at its spike protein, which carries 30 substitutions, 3 short deletions and 1 insertion. Among these changes, there were 8 and 15 mutations located at the N-terminal domain (NTD) and receptor-binding domain (RBD), respectively. In addition, several substitutions are located at or near the spike S_1_/S_2_ cleavage site. BA.2 spike shares 21 substitutions with that of BA.1 when compared to the ancestral SARS-CoV-2. Meanwhile, BA.2 spike carries 8 specific mutations, including 4 changes (T19I, L24S, 25–27deletion, V213G) in the NTD and 4 substitutions (S371F, T376A, D405N, R408S) in the RBD. BA.2.12.1 spike contains two substitutions, L452Q and S704L, when compared to BA.2. BA.4 and BA.5 share the same spike sequence, which contains three substitutions (L452R, F486V, R493Q) and the 69–70 deletion when compared with BA.2.

We and others recently demonstrated that Omicron BA.1 is less pathogenic compared to SARS-CoV-2 wildtype (WT) or previous variants of concerns (VOCs) [12–14], which is contributed by the unique virological features of Omicron BA.1 in comparison to other SARS-CoV-2 strains, including less efficient transmembrane serine protease 2 (TMPRSS2) usage, less spike cleavage, lower fusogenicity, and an altered entry mechanism [12,14–18]. More recent studies suggest that these virological features of Omicron BA.1 are in part retained by the subsequent Omicron sublineages [19–22]. However, the exact spike determinants that contribute to these observed virological features of Omicron and Omicron sublineages have not been extensively explored. Here, we evaluated the spike determinants for the observed virological characteristics of Omicron BA.1 and BA.2. Our study identified key changes in the spike that contributed to the lower TMPRSS2 usage, reduced spike cleavage, lower fusogenicity, and altered entry mechanism of Omicron BA.1 and BA.2. Together, these findings improved our current knowledge on the virological determinant and pathogenicity of Omicron and Omicron sublineages.

## Materials and methods

### Viruses and safety

SARS-CoV-2 HKU-001a (WT) (GenBank: MT230904), B.1.617.2/Delta (GenBank: OM212471) and B.1.1.529.1/Omicron BA.1 (GenBank: OM212472) were clinical isolate strains from laboratory-confirmed COVID-19 patients in Hong Kong [23,24]. SARS-CoV-2 HKU-001a S_1_/S_2_-10Del (GenBank: MT621560), which carries a 10 amino-acid deletion, was isolated by plaque purification from SARS-CoV-2 cultured in VeroE6 cells and subsequently sequenced [25]. SARS-CoV-2 HKU-001a, Delta and Omicron were cultured using VeroE6-TMPRSS2 cells and titrated by plaque assays [12,26]. All experiments with infectious SARS-CoV-2 were performed according to the approved standard operating procedures of the Biosafety Level 3 facility at Department of Microbiology, HKU [27].

### Cell cultures

293T and VeroE6 were obtained from ATCC and maintained in Dulbecco’s modified Eagle’s medium (DMEM) (11965-092, Gibco, Amarillo, Texas, USA) according to supplier’s instructions. Calu3 was obtained from ATCC and maintained in DMEM/F12 (11320-033, Gibco). VeroE6-TMPRSS2 was obtained from the Japanese Collection of Research Bioresources (JCRB) Cell Bank and cultured in DMEM. All cell lines used are routinely tested for mycoplasma and are maintained mycoplasma-free.

### Virus replication in cell lines

Calu3 and VeroE6 cells were challenged by WT, Delta, Omicron BA.1 and S_1_/S_2_-10Del at 0.5 MOI or 0.1 MOI. At 24 hpi, the cell lysates were harvested for qRT-PCR quantification of virus replication. Viral RNA from infected cells was extracted using QIAsymphony RNA Kit (931636, Qiagen, Germantown Road Germantown, MD, USA). Viral subgenomic RNA of E gene was quantified using the QuantiNova Probe RT–PCR Kit (208354, Qiagen) [28].

### Production of SARS-CoV-2-spike pseudoviruses

All mutated SARS-CoV-2-spike pseudoviruses were packaged as described previously [25,29]. In brief, 293T cells were transfected with different spikes, or VSV-G plasmids with Lipofectamine 3000 (L3000015, Thermo Fisher Scientific, Waltham, MA, USA). At 24 h post-transfection, the cells were transduced with VSV-deltaG-firefly pseudotyped with VSV-G. At 2 h post-transduction, the cells were washed three times with PBS and cultured in fresh media with an anti-VSV-G (8G5F11) antibody (EB0010, kerafast, Boston, MA, USA). The pseudoviruses were then harvested 16 h post-transduction and titrated with TCID_50_. All single mutation plasmids of SARS-CoV-2 spike were constructed in Genscript (Nanjing, China). Q498R was not included in the study because of its low expression.

### Protease usage assays

293T cells were transfected with hACE2 or co-transfected with hACE2 and different protease plasmids including TMPRSS2, TMPRSS11D, TMPRSS13, Cathepsin L or Cathepsin B plasmids. The transfected cells were inoculated with pseudoviruses for 24 h post-transfection and cultured in 1% FBS media for another 18 h, before washed and lysed for detection of luciferase signal with a luciferase assay system (E1501, Promega, Madison, WI, USA). The protease plasmids were obtained from OriGene (Rockville, MD, USA) or Sino Biological (Beijing, China).

### Western blot analysis of spike cleavage

Spike plasmids of SARS-CoV-2 D614G, Omicron BA.1, BA.2, S_1_/S_2_-10Del and all single mutation plasmids were transfected with Lipofectamine 3000 (L3000015, Thermo Fisher Scientific) in 293T cells. Cell lysates were harvested 24 h post-transfection for Western blot analysis. Specific primary antibodies were incubated with the blocked membranes at 4°C overnight, followed by horseradish peroxidase (HRP) conjugated secondary antibodies (62-6520, Thermo Fisher Scientific) for 1 h at room temperature. The signal was developed by SuperSignal West Pico PLUS Chemiluminescent Substrate (34580, Thermo Scientific, USA) and detected using Alliance Imager apparatus (Uvitec, Cambridge, UK). The full-length spike and S2 were detected with a rabbit anti-SARS-CoV-2 spike S2 antibody (40590-T62, Sino Biological) (1:5000). β-actin was detected with a β-actin antibody (clone AC-74, A5316, Sigma, USA) (1:5000). The cleavage ratio of the spike was quantified by ImageJ.

### Cell–cell fusion assay

293T cells were co-transfected with different SARS-CoV-2 spike plasmids with GFP1-10 plasmid (cat#68715, Addgene) as effector cells. Another population of 293T cells was co-transfected with human ACE2 (hACE2), TMPRSS2, and GFP11 (cat#68716, Addgene) as target cells. After 24 h post-transfection, the effector and target cells were digested by EDTA-Trypsin (25200072, Gibco) and mixed at a 1:1 ratio. The mixed cells were co-cultured at a 37°C incubator for another 24 h. The mixed cells were fixed in 10% formalin and then permeabilized with 0.1% Triton-X100 (11332481001, Sigma, USA) at room temperature. The antifade mounting medium with 4′,6-Diamidino-2-Phenylindole, Dihydrochloride (DAPI, H-1200, Vector Laboratories) was used for mounting and DAPI staining. Images were taken with the Olympus BX73 fluorescence microscope (Olympus Life Science, Tokyo, Japan). The fusion area of images was quantified by ImageJ.

### Protease inhibitor treatment assay

The serine protease inhibitor, camostat (HY-13512), and the cysteine protease inhibitor, E64D (HY-100229), were purchased from MedChemExpress (Monmouth Junction, NJ, USA). Calu3 or VeroE6-TMPRSS2 cells were treated with DMSO, Camostat, or E64D at concentrations of 1, 25, and 50 µM for 2 h before authentic virus infection. At 24 hpi, the cell lysates were harvested for qRT-PCR quantification of virus replication. For pseudovirus entry assays, VeroE6-TMPRSS2 cells were treated with DMSO, Camostat, or E64D at concentrations of 1, 25, and 50 µM for 2 h before pseudoviruses transduction. The cell lysates were lysed for detection of luciferase signal 18 h post-transduction.

### Statistical analysis

Statistical comparison among three or more experiment groups was performed with one-way ANOVA with Tukey’s multiple comparison test. Differences were considered statistically significant when *p* < 0.05. * represented *p* < 0.05, ** represented *p* < 0.01, *** represented *p* < 0.001, and **** represented *p* < 0.0001. ns, not statistically significant. Data analysis was performed with Graphpad prism 8.0.

## Results

### Omicron BA.1 is substantially less dependent on TMPRSS2-mediated plasma membrane entry pathway and is highly dependent on the endosomal entry pathway for virus entry

**Figure 1.**
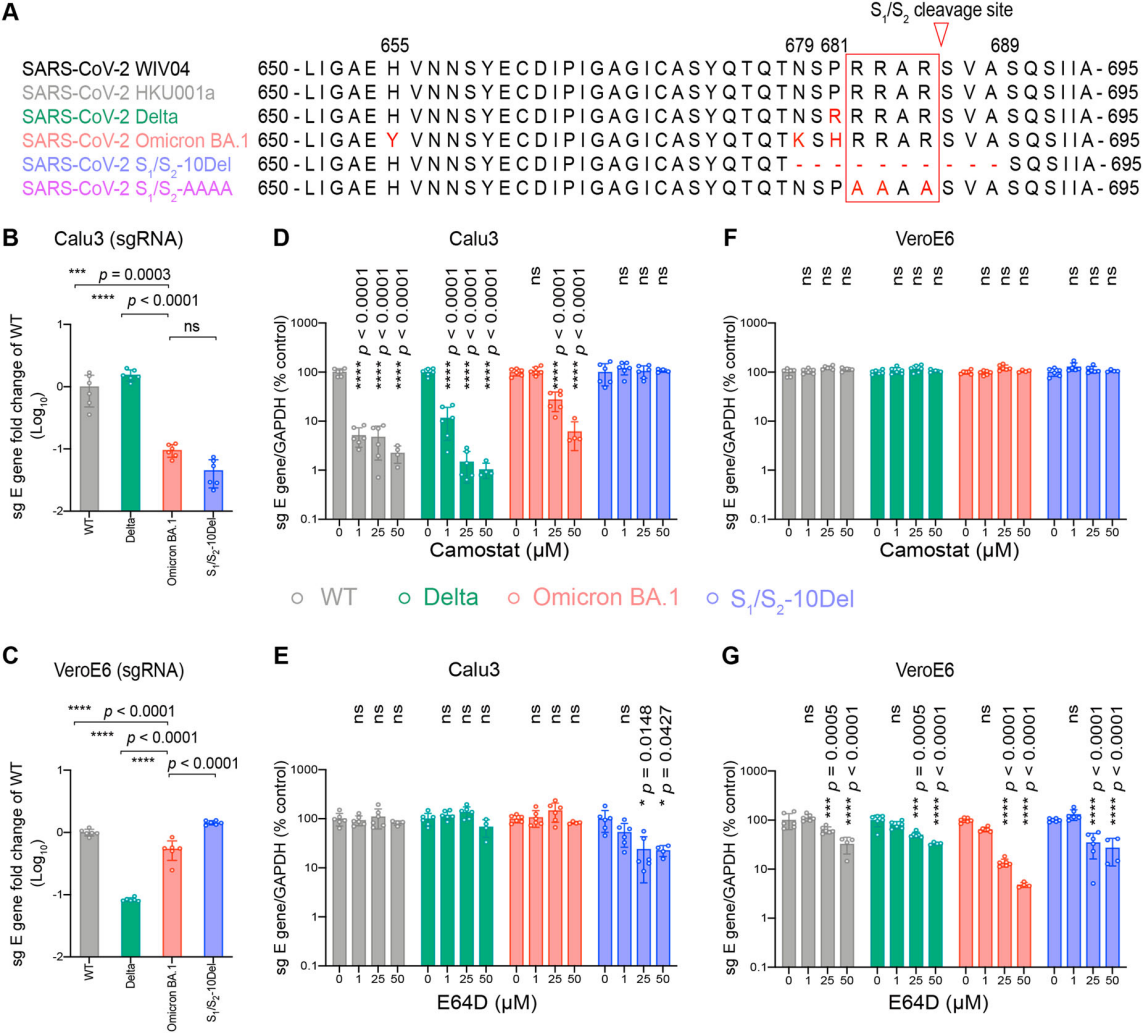
Omicron BA.1 is less dependent on TMPRSS2-mediated plasma membrane entry pathway and is highly dependent on the endosomal entry pathway for virus entry. (A) Amino-acid sequence alignment of residues around the S_1_/S_2_ cleavage site of SARS-CoV-2 reference strain WIV04, HKU001a, Delta, Omicron BA.1, S_1_/S_2_-10Del, and S_1_/S_2_-AAAA. Amino acid positions are designated based on SARS-CoV-2 reference strain. (B–C) Calu3 and VeroE6 cells were challenged with SARS-CoV-2 WT, Delta, Omicron BA.1, or S_1_/S_2_-10Del. Cell lysates were harvested at 24 hpi for quantification of the subgenomic RNA of the envelope (sgE) gene (*n* = 6). (D–G) Calu3 and VeroE6 cells were pre-treated with indicated concentrations of camostat or E64D for 2 h followed by the authentic SARS-CoV-2 variants infection. The amount of viral subgenomic envelope RNA in harvested cell lysates at 24 hpi was determined by qRT-PCR (*n *= 4 for 50uM of camostat and E64D groups, *n *= 6 for the other groups). Error bars were calculated by using log-transformed data and represented mean ± SD from the indicated number of biological repeats. Statistical significances were determined with one way-ANOVA. Data were obtained from three independent experiments. Each data point represents one biological repeat. * represented *p* < 0.05, ** represented *p* < 0.01, *** represented *p* < 0.001, and **** represented *p* < 0.0001. ns, not statistically significant; WT, wildtype SARS-CoV-2.

Ancestral SARS-CoV-2 infects lung cells predominantly through the TMPRSS2-mediated plasma membrane entry pathway [30–32]. In cells with low or no TMPRSS2 expression, ancestral SARS-CoV-2 can alternatively enter through endosomes mediated by endosomal proteases such as cathepsin L [33]. We and others recently demonstrated that SARS-CoV-2 Omicron BA.1 is less dependent on TMPRSS2 for virus entry [12,15,34]. In keeping with these findings, more recent reports have suggested an enhanced dependence of endosomal entry by Omicron BA.1 [18,35]. However, comprehensive analyses on Omicron BA.1 entry pathways in association with the contributing spike residues have not been carried out. To this end, we first evaluated the propensity of Omicron BA.1 in utilizing the plasma membrane entry pathway and the endosomal entry pathway for virus entry. For a more thorough investigation, we included SARS-CoV-2 WT, Delta, and S_1_/S_2_-10Del as comparison groups (Figure 1(A)). The Delta variant is highly efficient in using TMPRSS2-mediated plasma membrane entry pathway due to the P681R substitution in spike [36–39]. In contrast, the S_1_/S_2_-10Del isolate contained a 10 amino acid deletion flanking the S_1_/S_2_ cleavage site, resulting in substantially reduced efficiency in plasma membrane entry but gained efficiency in endosomal entry [25,30,40–42]. We first infected Calu3 and VeroE6 cells, which are model cell types for plasma membrane entry and endosomal entry, respectively, with SARS-CoV-2 WT, Delta, Omicron BA.1, and S_1_/S_2_-10Del, and quantified virus replication at 24 h post infection (hpi). In Calu3 cells, Omicron BA.1 replication was attenuated when compared to that of WT (*P *= 0.0003) and Delta (*P *< 0.0001), and was at similar level with that of S_1_/S_2_-10Del (*P *= ns) (Figure 1(B)). In VeroE6 cells, Omicron BA.1 replication was more efficient than that of Delta (*P *< 0.0001) but was lower when compared with that of WT (*P *< 0.0001) and S_1_/S_2_-10Del (*P *< 0.0001) (Figure 1(C)). These results indicate that Omicron BA.1 is less efficient in plasma membrane entry while is capable of efficient endosomal entry.

Next, we analyzed the dependency of SARS-CoV-2 WT, Delta, Omicron BA.1, and S_1_/S_2_-10Del on the plasma membrane entry pathway and the endosomal entry pathway by treating the infected cells with camostat (a serine protease inhibitor that inhibits plasma membrane entry) or E64D (a cysteine protease inhibitor that inhibits endosomal entry). In Calu3 cells, camostat treatment inhibited the replication of WT, Delta, and Omicron BA.1 in a dose-dependent manner, but not S_1_/S_2_-10Del. In keeping with previous findings [12,15], Omicron BA.1 was less sensitive to camostat inhibition as 1μM camostat reduced WT and Delta replication by 94.9% (*P *< 0.0001) and 88.3% (*P *< 0.0001), respectively, but did not reduce Omicron BA.1 replication (*P *= ns) (Figure 1(D)). At high camostat concentration, 50μM camostat reduced WT, Delta, and Omicron replication to 2.25% (*P* < 0.0001), 1.04% (*P* < 0.0001), and 6.11% (*P* < 0.0001) when compared to that of their controls, suggesting that Omicron BA.1 is 2.7-folds and 5.9-folds less sensitive than WT and Delta, respectively, at this high camostat concentration (Figure 1(D)). E64D treatment in Calu3 reduced the replication of S_1_/S_2_-10Del, but not that of WT, Delta, and Omicron BA.1 (Figure 1(E)). In VeroE6 cells, camostat treatment did not reduce the replication of all evaluated viruses since this cell type is deficient in TMPRSS2 expression (Figure 1(F)). Meanwhile, E64D treatment in VeroE6 cells most substantially inhibited the replication of Omicron BA.1 among all evaluated viruses (Figure 1(G)). 50μM E64D reduced the replication of WT, Delta, Omicron BA.1, and S_1_/S_2_-10Del by 57.7% (*P *< 0.0001), 65.1% (*P *< 0.0001), 95.2% (*P *< 0.0001), and 72.9% (*P *< 0.0001), respectively (Figure 1(G)). Collectively, these results indicate that Omicron BA.1 is substantially less dependent on TMPRSS2-mediated plasma membrane entry pathway and is highly dependent on the endosomal entry pathway for virus entry.

### Spike determinants for the reduced TMPRSS2 usage of Omicron BA.1 and BA.2

**Figure 2.**
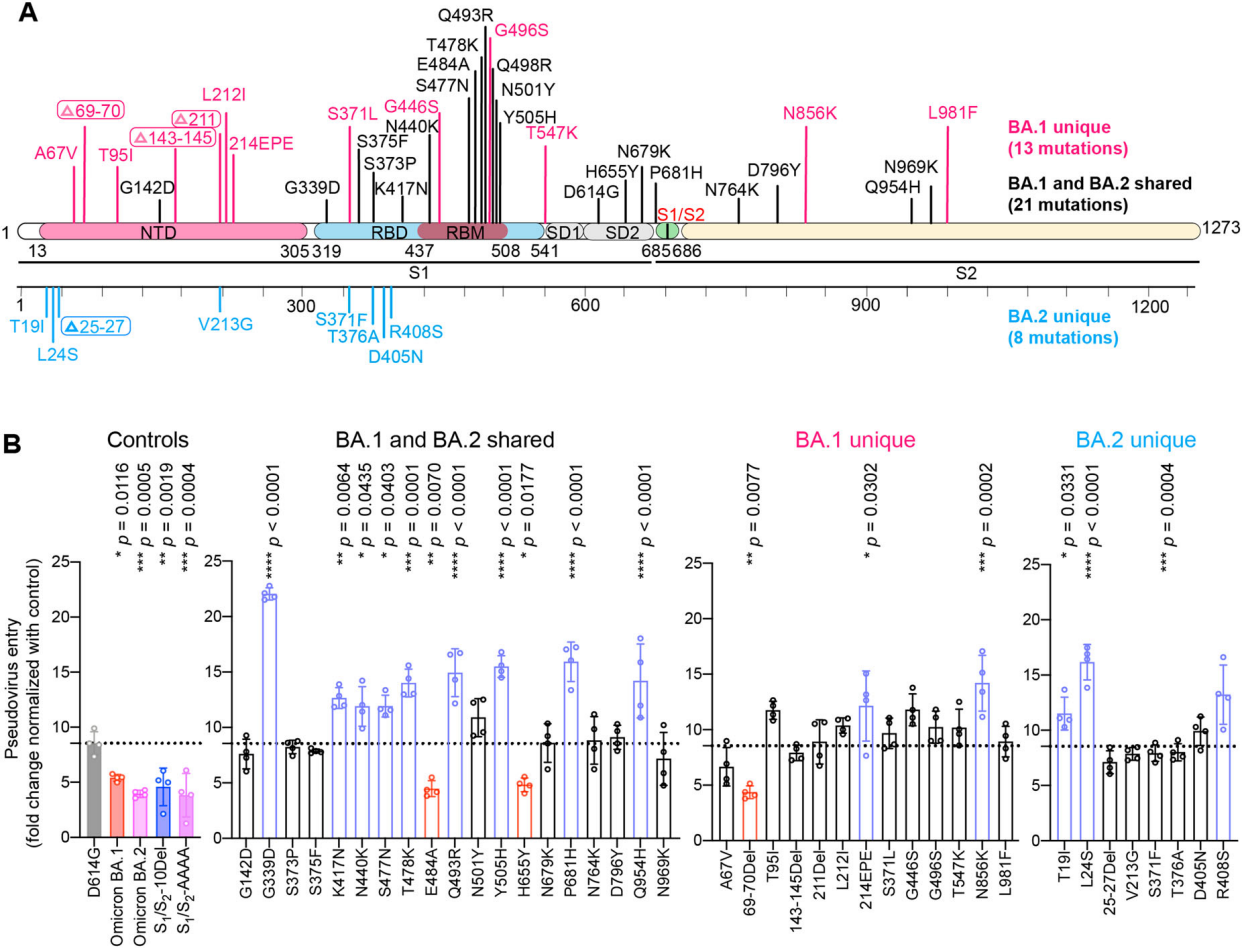
Spike determinants for the reduced TMPRSS2 usage of Omicron BA.1 and BA.2. (A) Schematic of all amino acid mutation sites on Omicron BA.1 and BA.2 spike when compared to ancestral SARS-CoV-2 spike. (B) 293T cells were transfected with hACE2 or co-transfected with hACE2 and TMPRSS2, followed by transduction with pseudoviruses bearing the spike of SARS-CoV-2 D614G, Omicron BA.1, Omicron BA.2, S_1_/S_2_-10Del, S_1_/S_2_-AAAA and individual mutation at 24 h post-transfection. Pseudovirus entry was quantified by measuring the luciferase signal (*n *= 4). Fold changes in the luciferase signal were normalized to the mean luciferase readouts of cells with only hACE2 overexpression. Data represent mean ± SD from the indicated number of biological repeats. Statistical significance was determined with one way-ANOVA. Data were obtained from three independent experiments. Each data point represents one biological repeat. * represented *p* < 0.05, ** represented *p* < 0.01, *** represented *p* < 0.001, and **** represented *p* < 0.0001. ns, not statistically significant.

Recent studies identified altered virological features of Omicron BA.1 in comparison to SARS-CoV-2 WT and previous variants, including less efficient TMPRSS2 usage, less spike cleavage, and lower fusogenicity, which may explain its altered entry mechanism and change in pathogenicity [12–15,18,34]. Additional studies suggest that these virological features of Omicron BA.1 are in part retained by the subsequent Omicron sublineages, including BA.2 [19–22]. Omicron BA.1 spike carries 34 changes when compared to the ancestral SARS-CoV-2 spike. Omicron BA.2 spike shares 21 substitutions with that of Omicron BA.1 when compared to the ancestral SARS-CoV-2. Meanwhile, Omicron BA.2 spike carries 8 specific mutations in its NTD and RBD (Figure 2(A)). To delineate the specific spike changes that contribute to the observed virological features in Omicron BA.1 and BA.2, we constructed vesicular stomatitis virus (VSV)-based SARS-CoV-2-spike pseudoviruses carrying individual mutations present on Omicron BA.1 and BA.2 spike with the D614G background. First, we compared the efficiency of TMPRSS2 usage of this panel of pseudoviruses with Omicron BA.1-, BA.2-, S_1_/S_2_-10Del-, and S_1_/S_2_-AAAA-pseudoviruses included as controls. S_1_/S_2_-AAAA was an additional control that contained three amino acid changes (R682A, R683A and R685A) that changed the S_1_/S_2_ multibasic cleavage site from RRAR [43] to AAAA (Figure 1(A)). We transfected 293T cells with hACE2 or hACE2 and TMPRSS2, followed by transducing the cells with the panel of pseudoviruses and quantified virus entry at 18 h post transduction. Our results showed that TMPRSS2 overexpression increased D614G-pseudovirus entry by 8.54-fold. In keeping with previous literature [12,15,30,41], Omicron BA.1-, Omicron BA.2-, S_1_/S_2_-10Del-, and S_1_/S_2_-AAAA-pseudoviruses were attenuated in TMPRSS2 usage in comparison to that of D614G-pseudoviruses. Importantly, our results revealed three spike changes that significantly attenuated TMPRSS2 usage when compared to that of D614G, including 69-70Del (4.35-fold; *P *= 0.0077), E484A (4.47-fold; *P *= 0.0070), and H655Y (4.82-fold; *P *= 0.0177) (Figure 2(B)). Interestingly, these three changes facilitated ACE2-mediated entry into 293T cells (Supplementary Figure 1). Since 69-70Del is only present in Omicron BA.1 but not BA.2 spike, our results indicate that changes at E484A and H655Y contribute to the reduced TMPRSS2 usage of Omicron BA.1 and BA.2.

### Spike determinants for the reduced spike cleavage of Omicron BA.1 and BA.2

**Figure 3.**
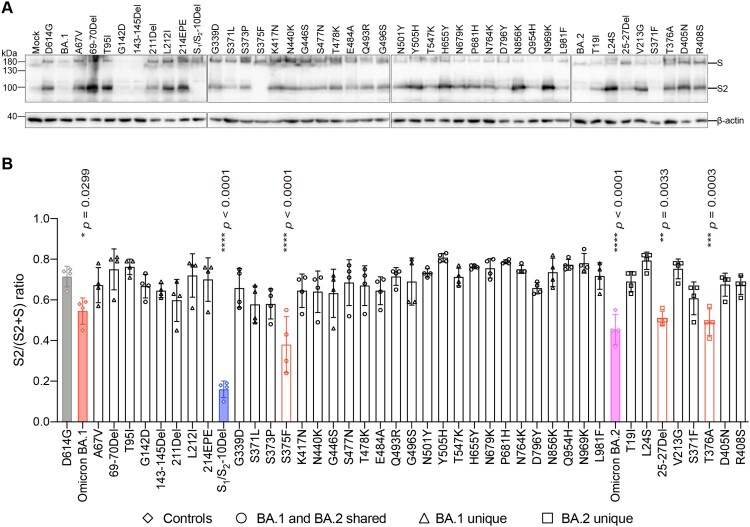
Spike determinants for the reduced spike cleavage of Omicron BA.1 and BA.2. (A) 293T cells were transfected with the indicated spike plasmids. Cell lysates were harvested at 24 h post-transfection for detection of SARS-CoV-2 spike cleavage using an anti-spike S2 antibody. Representative images of spike were shown with β-actin added as a sample processing control. Spike and β-actin were run on different gels and detected on different membranes. The experiment was repeated four times independently with similar results. (B) The cleavage ratio of different spikes from four times independent experiments was quantified by ImageJ. Data represent mean ± SD from the indicated number of biological repeats. Statistical significance was determined with one way-ANOVA. Data were obtained from four independent experiments. Each data point represents one biological repeat. * represented *p* < 0.05, ** represented *p* < 0.01, *** represented *p* < 0.001, and **** represented *p* < 0.0001. ns, not statistically significant.

Omicron BA.1 and BA.2 spike carry two substitutions at the S_1_/S_2_ cleavage site (N679K, P681H) and three substitutions near the S_1_/S_2_ cleavage site (H655Y, N764K, D796Y) (Figure 2(A)). Previous reports suggested that mutations in the N-terminal domain (NTD) and receptor-binding domain (RBD) of spike can also alter spike cleavage [44–46]. To investigate the spike mutation that may contribute to the reduced spike cleavage of Omicron BA.1 and BA.2, we transfected 293T cells with the panel of spike constructs and harvested cell lysates at 24 h post transfection for Western blot analysis. Our results revealed that the cleavage of Omicron BA.1, BA.2, and S_1_/S_2_-10Del spike was significantly reduced in comparison that of D614G spike (Figure 3(A,B)), in keeping with previous reports [12,14,15,34,47]. Intriguingly, among the panel of spike constructs, the 25-27Del, S375F, and T376A changes significantly reduced spike cleavage when compared to that of D614G spike (Figure 3(A,B)). Since 25-27Del and T376A are present only in Omicron BA.2 but not in Omicron BA.1 spike, our results indicate that the reduced spike cleavage detected in Omicron BA.1 and BA.2 is associated with the S375F substitution.

### Determinants for the reduced spike-mediated cell–cell fusion of Omicron BA.1 and BA.2

**Figure 4.**
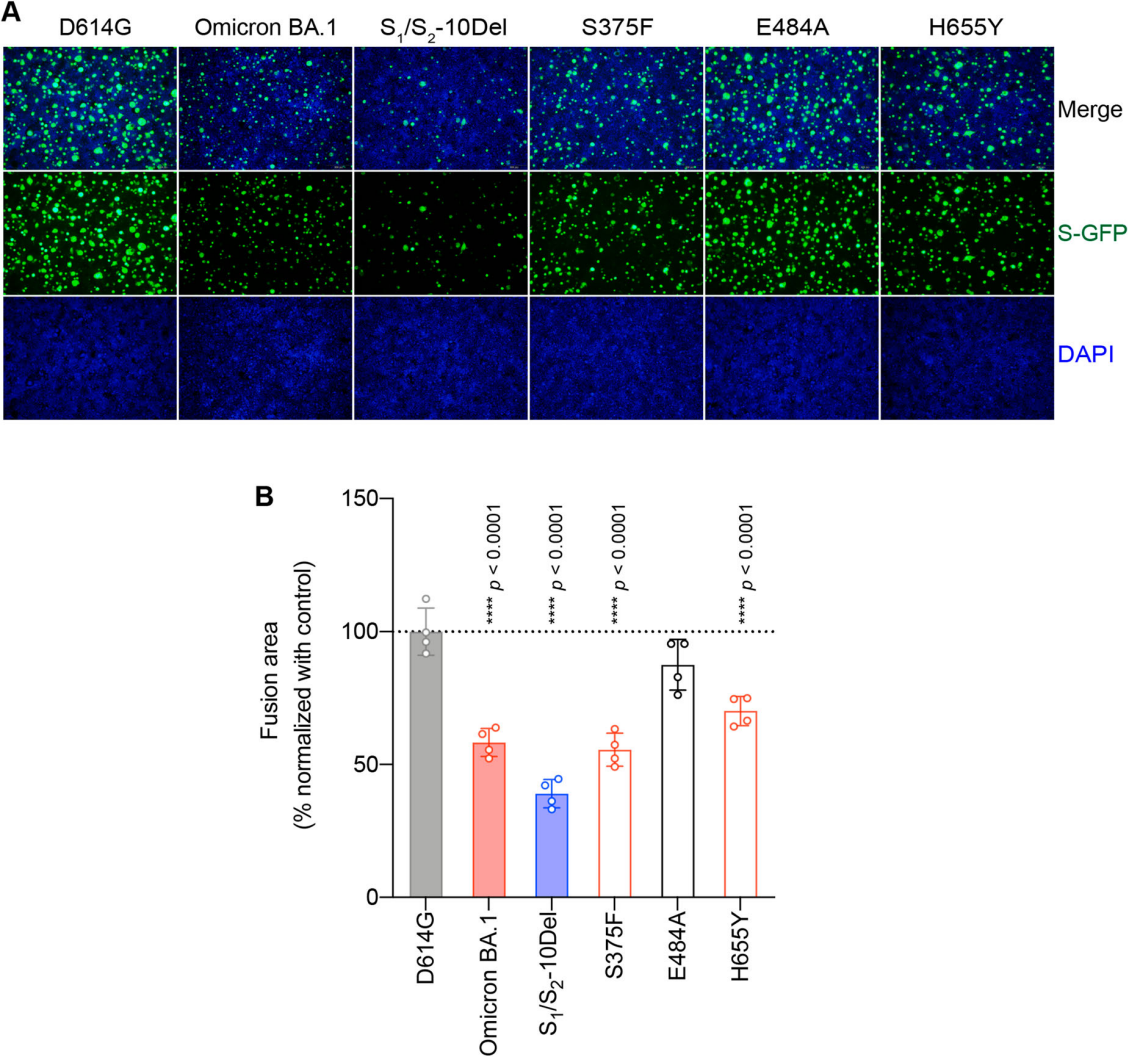
Determinants for the reduced spike-mediated cell–cell fusion of Omicron BA.1 and BA.2. (A) Representative images of spike-mediated cell–cell fusion. 293T cells (effectors cells) were co-transfected with the indicated spike with GFP1-10, and were co-cultured with 293T cells co-transfected with human ACE2 (hACE2), TMPRSS2, and GFP11 (target cells). The co-cultured cells were fixed with 10% formalin and stained with DAPI. Representative images were from four independent experiments with similar results. (B) The fusion area was normalized with the SARS-CoV-2-D614G spike-mediated cell–cell fusion group by ImageJ. Data represent mean ± SD from the indicated number of biological repeats. Statistical significance was determined with one way-ANOVA. Data were obtained from four independent experiments. Each data point represents one biological repeat. * represented *p* < 0.05, ** represented *p* < 0.01, *** represented *p* < 0.001, and **** represented *p* < 0.0001. ns, not statistically significant.

Next, we evaluated the spike mutations that may contribute to the reduced spike-mediated cell–cell fusion of Omicron BA.1 and BA.2. To this end, we analyzed spike-mediated cell–cell fusion assays in 293T cells using the split GFP system [48–50]. We focused our analysis on S375F, E484A, and H655Y since these changes are shared by both Omicron BA.1 and BA.2, and contributed to either reduced TMPRSS2 usage or reduced spike cleavage. Our results suggested that cell–cell fusion induced by Omicron BA.1 and S_1_/S_2_-10Del spike was significantly reduced in comparison to that of D614G spike (Figure 4(A,B)), in keeping with previous reports [14,15,30]. In addition, we found that spike-mediated cell–cell fusion was significantly attenuated for S375F (*P *< 0.0001) and H655Y (*P *< 0.0001), suggesting that these mutations are associated with the decreased fusogenicity of Omicron BA.1 and BA.2 spike.

### Spike determinants for the altered entry mechanism of Omicron BA.1 and BA.2

**Figure 5.**
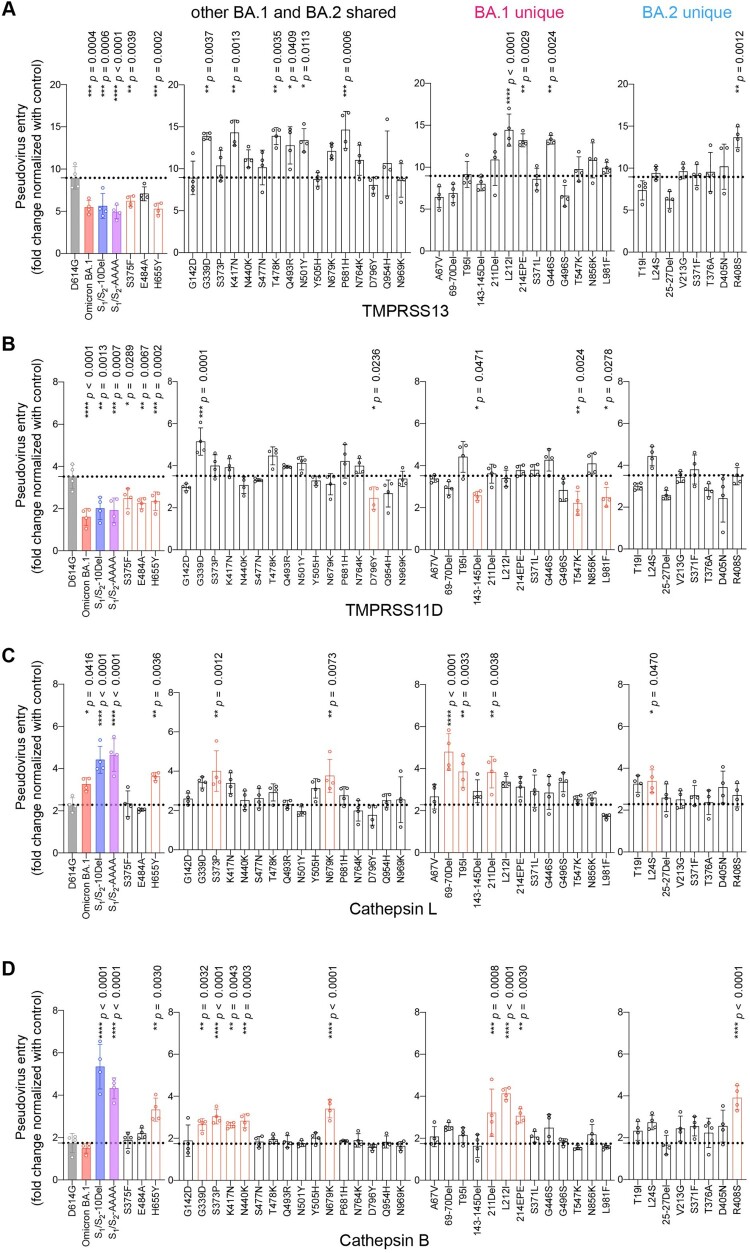
Spike determinants for the altered entry mechanism of Omicron BA.1 and BA.2. A-D. 293T cells were transfected with hACE2 or co-transfected with hACE2 and TMPRSS13 (A), TMPRSS11D (B), Cathepsin L (C), or Cathepsin B (D), followed by transduction with pseudoviruses expressing the indicated spike at 24 h post-transfection. Pseudovirus entry was quantified by measuring the luciferase signal (*n *= 4). Fold changes in the luciferase signal were normalized to the mean luciferase readouts of cells with only hACE2 overexpression. Data represent mean ± SD from the indicated number of biological repeats. Statistical significance was determined with one way-ANOVA. Data were obtained from three independent experiments. Each data point represents one biological repeat. * represented *p* < 0.05, ** represented *p* < 0.01, *** represented *p* < 0.001, and **** represented *p* < 0.0001. ns, not statistically significant.

Recent evidence revealed that additional transmembrane serine proteases TMPRSS11D and TMPRSS13 can similarly activate SARS-CoV-2 spike and facilitate SARS-CoV-2 spike pseudovirus entry at the plasma membrane [51–53]. Besides the plasma membrane pathway, SARS-CoV-2 alternatively enters through the endosomal pathway where the spike proteins on virus particles are activated by cathepsin L or cathepsin B in the endosomes [30,31,33]. However, whether or not TMPRSS11D, TMPRSS13, cathepsin L, and cathepsin B are differentially involved in Omicron BA.1 entry is currently incompletely understood. To this end, we first evaluated TMPRSS11D and TMPRSS13 on their capacities to mediate Omicron BA.1 pseudovirus entry. We found that Omicron BA.1 pseudovirus utilized TMPRSS11D and TMPRSS13 at a decreased efficiency when compared to SARS-CoV-2 D614G pseudovirus, and was at comparable level with that of S_1_/S_2_-10Del and S_1_/S_2_-AAAA pseudoviruses (Figure 5(A,B)). In keeping with our findings described earlier, changes at S375F, E484A, and H655Y reduced TMPRSS11D and/or TMPRSS13 usage, with S375F and H655Y consistently reduced both TMPRSS11D and TMPRSS13 usage (Figure 5(A,B)). Next, we evaluated the role of cathepsin L and cathepsin B on their capacities to facilitate Omicron BA.1 pseudovirus entry and we found that cathepsin L but not cathepsin B promoted Omicron BA.1 pseudovirus entry at an increased efficiency over that of SARS-CoV-2 D614G pseudovirus (Figure 5(C,B)). Interestingly, both cathepsin L and cathepsin B promoted the entry of S_1_/S_2_-10Del and S_1_/S_2_-AAAA pseudoviruses at significantly increased efficiency when compared with SARS-CoV-2 D614G pseudoviruses (Figure 5(C,D)). Under this setting, the H655Y substitution consistently increased both cathepsin L and cathepsin B usage (Figure 5(C,D)). In parallel, we extended our analysis to the entire panel of Omicron BA.1 and BA.2 individual mutations. We found that although a number of the spike mutations reduced TMPRSS13/11D usage while others increased cathepsin L/B usage, the H655Y substitution was the one that consistently altered serine protease (TMPRSS2, TMPRSS11D, TMPRSS13) and cysteine protease (cathepsin L and cathepsin B) usage (Figures 2(B) and 5(A–D)).

**Figure 6.**
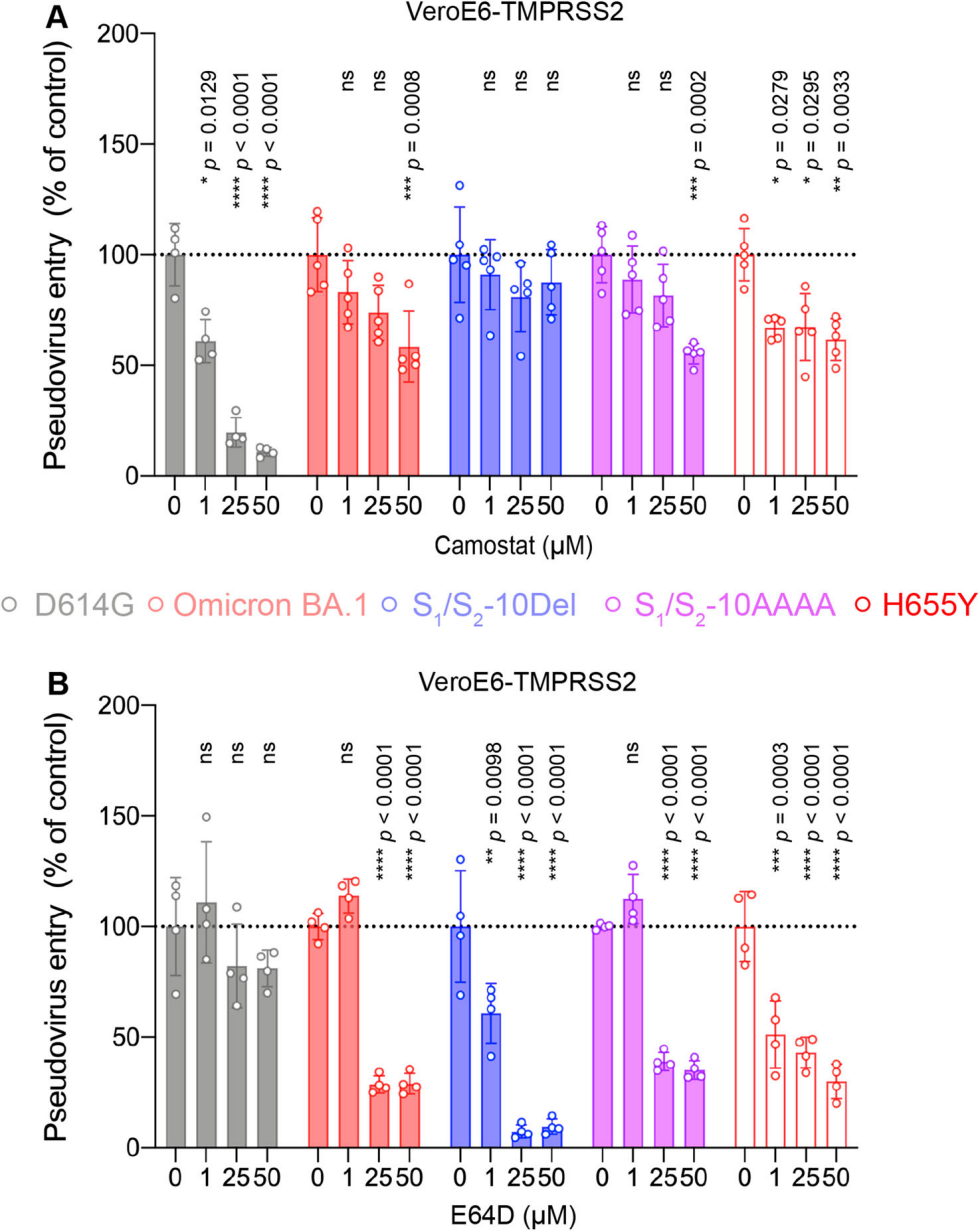
The H655Y substitution in Omicron BA.1 and BA.2 spike promotes endosomal entry. (A–B) VeroE6-TMPRSS2 cells were pre-treated with 1, 25, or 50μM camostat (A) or E64D (B) or DMSO (A–B) for 2 h followed by transduction with pseudoviruses expressing the indicated spike at 24 h post-transfection. Pseudovirus entry was quantified by measuring the luciferase signal (*n *= 5 for camostat treatment of Omicron BA.1, S_1_/S_2_-10Del, S_1_/S_2_-AAAA, and H655Y; *n* = 4 for the other groups). Data represent mean ± SD from the indicated number of biological repeats. Statistical significances were determined with one way-ANOVA. Data were obtained from three independent experiments. Each data point represents one biological repeat. * represented *p* < 0.05, ** represented *p* < 0.01, *** represented *p* < 0.001, and **** represented *p* < 0.0001. ns, not statistically significant.

We next asked if H655Y plays a role in modulating the entry pathways utilized by Omicron BA.1. To this end, we compared SARS-CoV-2 D614G, Omicron BA.1, S_1_/S_2_-10Del, S_1_/S_2_-AAAA, and H655Y pseudovirus entry in VeroE6-TMPRSS2 cells, which supports both plasma membrane entry and endosomal entry, in the presence of camostat or E64D. Our results demonstrated that 50μM camostat reduced SARS-CoV-2 D614G pseudovirus entry by 89.11% (*P *< 0.0001) while 50μM E64D did not significantly reduce SARS-CoV-2 D614G pseudovirus entry (*P *= ns) (Figure 6(A,B)). In sharp contrast, 50μM camostat only reduced H655Y pseudovirus entry by 34.81% (*P *= 0.0033) while 50μM E64D significantly reduced H655Y pseudovirus entry by 69.99% (*P *< 0.0001) (Figure 6(A,B)). These results indicated that the H655Y pseudoviruses are more dependent on the endosomal entry pathway for virus entry, which are similar to Omicron BA.1, S_1_/S_2_-10Del, and S_1_/S_2_-AAAA pseudoviruses, and are different from the SARS-CoV-2 D614G pseudovirus (Figure 6(A,B)).

## Discussion

Omicron BA.1 and BA.2 carry several unique features that distinguish it from the ancestral SARS-CoV-2 or other previous VOCs [12–17]. These features, including the less efficient TMPRSS2 usage, less spike cleavage, lower fusogenicity, and altered entry mechanism together contributed to its lower pathogenicity in animal models and humans. As of today, the specific amino acid changes contributing to each of these observed phenotypes remain incompletely understood. In this study, we investigated on the spike determinants that contributed to these important virological features of Omicron BA.1 and BA.2. By screening each individual change on Omicron BA.1 and BA.2 spike, we identified that 69–70 deletion, E484A, and H655Y contributed to reduced TMPRSS2 usage while 25–27 deletion, S375F, and T376A resulted in less efficient spike cleavage. Among the shared mutations of BA.1 and BA.2 spike, S375F and H655Y reduced fusogenicity. Interestingly, the H655Y change consistently reduced serine protease (TMPRSS2, TMPRSS13, TMPRSS11D) usage while increased the use of endosomal proteases (cathepsin L and cathepsin B). In keeping with these findings, the H655Y substitution alone reduced plasma membrane entry and facilitated endosomal entry when compared to SARS-CoV-2 WT. Overall, our study identified key changes in Omicron BA.1 and BA.2 spike that contribute to our current knowledge on the virological determinant and pathogenicity of Omicron and Omicron sublineages.

SARS-CoV-2 spike is first processed during virus egress by furin at the S_1_/S_2_ site [41,54–56]. Upon binding host angiotensin-converting enzyme 2 (ACE2) on target cells, the pre-processed spike will be further cleaved by TMPRSS2 or other transmembrane serine proteases to facilitate virus fusion with the plasma membrane [51–53,57,58]. Interestingly, in the current study, the identified amino acid changes (25–27 deletion, 69–70 deletion, S375F, T376A, E484A, H655Y) that modulated Omicron BA.1/BA.2 entry-related events, including spike cleavage and TMPRSS2 usage, were not located at the S_1_/S_2_ site. In this regard, the exact mechanisms of how these amino acid changes dictate Omicron BA.1 and BA.2 entry warrant further investigation. The findings of our study are in keeping with that from a recent preprint, which suggested the spike S375F substitution could attenuate spike cleavage and fusogenicity due to an interprotomer pi-pi interaction with the H505 residue in another protomer in the spike trimer [45]. A separate preprint similarly suggested the contribution of the spike H655Y substitution in the altered capacity of endosomal entry of Omicron BA.1 without modulating BA.1 spike cleavage [16]. In contrast, a previous report suggested that the H655Y substitution in a clinical isolate could increase spike cleavage and fusion, leading to more efficient virus replication and transmission [59]. The reason behind the discrepancy on the role of H655Y between these studies is currently unknown but may in part attribute to the additional mutations present in the clinical isolate. Alternatively, spike cleavage may be differentially presented in virus-infected and spike-overexpressed cells. The pattern of spike cleavage may also differ based on the models of investigation since the endogenous expression of spike-processing proteases may be different. Importantly, H655Y is also carried by the Gamma variant (P.1), yet Gamma is not known to demonstrate the entry preference of Omicron BA.1 and BA.2. In this regard, the combined effect of the changes on Omicron BA.1 and BA.2 spike should be further evaluated.

Our study has a number of limitations. First, the current study focused on single spike mutations. It is possible that combined analysis of two or more spike mutations is important to better understand the TMPRSS2 usage, spike cleavage, and cell–cell fusion of Omicron. Second, the findings of the current study should be further verified in the context of infectious recombinant viruses.

Overall, by systemically screening the individual mutations on Omicron BA.1 and BA.2 spike, our study reveals key determinants in Omicron BA.1 and BA.2 spike that contribute to our understanding on the virological determinant and pathogenicity of Omicron and Omicron sublineages.

## Supplementary Material

Supplemental Material
