# Supplementary figures and images for: Spike mutations contributing to the altered entry preference of SARS-CoV-2 omicron BA.1 and BA.2
Source: Emerg Microbes Infect. 2022 Sep 28;11(1):2275–87. doi: 10.1080/22221751.2022.2117098 (PMC9542985; doi:10.1080/22221751.2022.2117098)

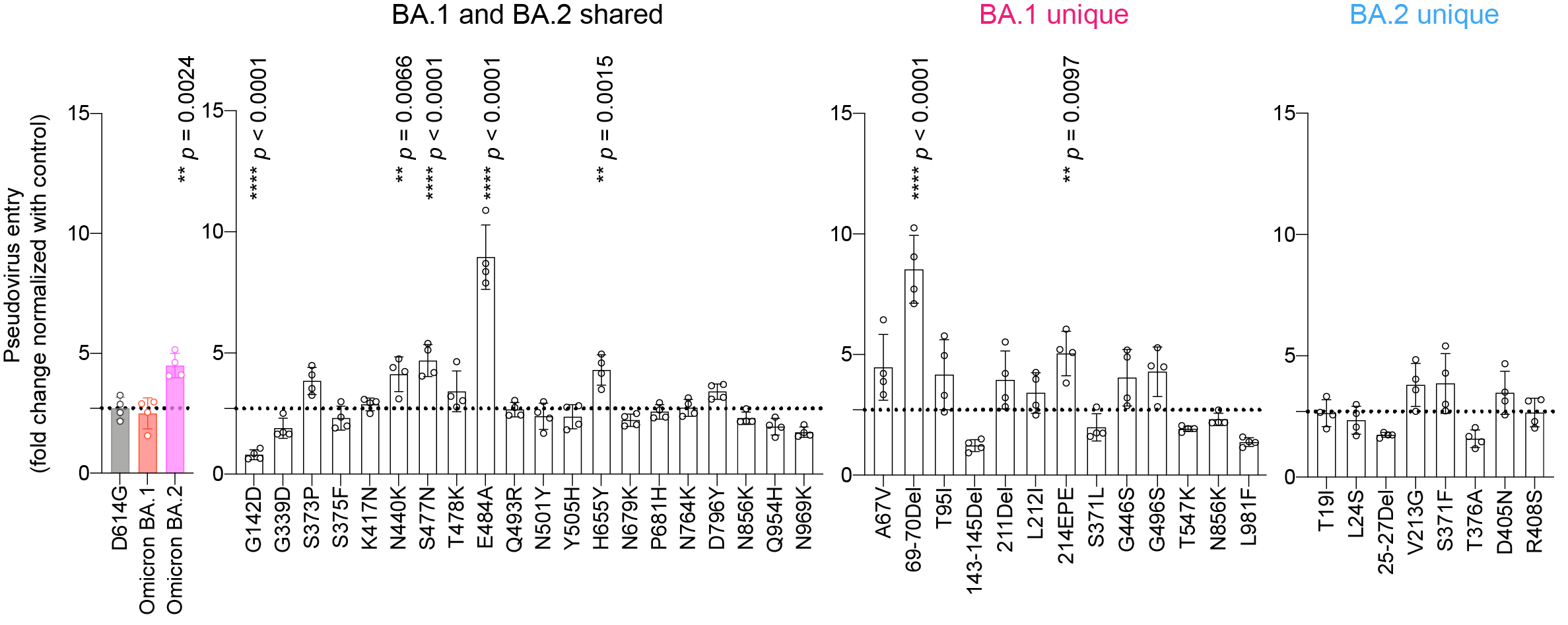

Supplement: Supplemental Material [file TEMI_A_2117098_SM8322.png]
